# Early Prediction in Classification of Cardiovascular Diseases with Machine Learning, Neuro-Fuzzy and Statistical Methods

**DOI:** 10.3390/biology12010117

**Published:** 2023-01-11

**Authors:** Osman Taylan, Abdulaziz S. Alkabaa, Hanan S. Alqabbaa, Esra Pamukçu, Víctor Leiva

**Affiliations:** 1 Department of Industrial Engineering, Faculty of Engineering, King Abdulaziz University, Jeddah 21589, Saudi Arabia; 2 University Medical Services Center, King Abdulaziz University, Jeddah 21589, Saudi Arabia; 3 Department of Statistics, Firat University, 23119 Elazığ, Turkey; 4 School of Industrial Engineering, Pontificia Universidad Católica de Valparaíso, Valparaíso 2362807, Chile

**Keywords:** adaptive neuro-fuzzy inference system, artificial intelligence, bioinformatics, cardiovascular diseases, classification, elastic net, myocardial infarction, statistical methods

## Abstract

**Simple Summary:**

Timely and accurate detection of cardiovascular diseases is critical to reduce the risk of myocardial infarction. This article proposes a methodology using machine learning, neuro-fuzzy and statistical methods to predict cardiovascular diseases. Our results show that the proposed methodology outperformed well known approaches, reaching a high prediction accuracy greater than 90%. Our methodology helps medical doctors to enhance diagnosis, quality of healthcare and efficacious prescriptions, decreasing the time for exams and minimizing expenses in clinical practice.

**Abstract:**

Timely and accurate detection of cardiovascular diseases (CVDs) is critically important to minimize the risk of a myocardial infarction. Relations between factors of CVDs are complex, ill-defined and nonlinear, justifying the use of artificial intelligence tools. These tools aid in predicting and classifying CVDs. In this article, we propose a methodology using machine learning (ML) approaches to predict, classify and improve the diagnostic accuracy of CVDs, including support vector regression (SVR), multivariate adaptive regression splines, the M5Tree model and neural networks for the training process. Moreover, adaptive neuro-fuzzy and statistical approaches, nearest neighbor/naive Bayes classifiers and adaptive neuro-fuzzy inference system (ANFIS) are used to predict seventeen CVD risk factors. Mixed-data transformation and classification methods are employed for categorical and continuous variables predicting CVD risk. We compare our hybrid models and existing ML techniques on a CVD real dataset collected from a hospital. A sensitivity analysis is performed to determine the influence and exhibit the essential variables with regard to CVDs, such as the patient’s age, cholesterol level and glucose level. Our results report that the proposed methodology outperformed well known statistical and ML approaches, showing their versatility and utility in CVD classification. Our investigation indicates that the prediction accuracy of ANFIS for the training process is 96.56%, followed by SVR with 91.95% prediction accuracy. Our study includes a comprehensive comparison of results obtained for the mentioned methods.

## 1. Introduction and Objectives

### 1.1. Introduction and Bibliographical Review

Cardiovascular diseases (CVDs) are related to arrhythmia, blood vessel problems, heart failure, myocardial infarction, strokes and other cardiac issues, these being some of the leading causes of death in the world [1]. In 2019, as reported by some organizations [2], over 17 million persons died from these diseases, which is more than 30% of all deaths worldwide during the same year. Thus, in healthcare, particularly for CVDs, timely and accurate detection of diseases and determining the vital risk factors are critically important.

Several risk prediction algorithms have been recommended, mainly employing regression methods integrating the data via well known risk factors [3]. However, these methods neglect the complexity and nonlinear characteristics of risk factors that have little or no mutual interaction [4], seriously affecting the prediction of CVDs.

CVDs include factors associated with blood pressure, cholesterol level, glucose level, living style and smoking, which can be controlled by taking medication and certain precautions. Nonetheless, factors such as age, ethnicity and family history of CVDs do not change with medication. Therefore, many factors need to be considered for accurate prediction of CVDs considering complexity and nonlinearity, justifying the use of artificial intelligence (AI) tools, which aid in predicting and classifying CVDs.

AI, machine learning (ML) and fuzzy logic play a vital role in the medical sciences to diagnose numerous diseases effectively in patients. ML is an advanced tool that allows systems to learn and improve automatically based on the experience of the target system. Supervised and unsupervised ML algorithms are the most common algorithms. A supervised ML algorithm uses the system’s past knowledge for new data. However, unsupervised ML algorithms utilize unclassified and unlabeled raw data.

ML techniques are reliable and efficient for predicting CVDs rather than naive ML and regression methods [5,6,7]. Several ML algorithms have been proposed during the last decade for forecasting CVDs using different parameters, datasets and approaches. For instance, ML approaches were proposed for predicting CVDs using the body mass index (BMI) [8]. Different ML models, such as decision trees, support vector machines (SVMs), artificial neural networks (ANNs), naive Bayes (NB) and random forests, were utilized to diagnose CVDs [9]. Among these models, the ANNs showed the best accuracy at 84.25%.

The mental load and other effects were statistically assessed with the analysis of variance test (ANOVA) using electroencephalogram (EEG) signals of thirty volunteer persons [10]. The predictive ability of ML algorithms using CVD data was evaluated in [11]. These authors found that SVM might outperform others when the objective is to maximize a specific mathematical function concerning the given dataset. Similarly, the ability of ML methods was compared, discovering that the SVM can identify hidden patterns in complex medical data [12]. Several researchers employed ML methods to validate their prediction frameworks. For instance, ML methods were utilized to obtain new insights on a dataset of over 40,000 patients with heart failure in Sweden [13]. These authors employed cluster analysis to discover four new illness phenotypes in this group.

Medical data of many UK patients were used to conduct prospective cohort research, which allowed a CVD incident to be predicted for ten years utilizing four ML approaches [14]. A random forests method was conducted to compare the conventional CVD risk ratings and to detect how well they might predict the six CVD events, considering participants of the MESA study [15]. The authors utilized the recommendations of some organizations (such as the Cardiology American College) that the growing number of patients potentially benefit from preventative medication using ML approaches.

When determining the effectiveness of algorithms, their accuracy level often matches that of other classifiers (as NB, logistic models and SVM). For instance, ten different factors concerning heart diseases in patients from South Africa and three different methods (NB, SVM and decision tree) were used to evaluate the approaches [16]. The Framingham scoring method was employed for risk classification of acute coronary heart diseases [17].

Deep learning algorithms were employed, with the k-nearest neighbor algorithm being identified as the better one with 66.7% accuracy rate compared to the random forest algorithm with 63.49% [15]. Thirteen factors and a collective CVD dataset were utilized to predict heart valve diseases and achieved a 92.0% accuracy rate [18]. ML algorithms based on their accuracies and computation time were applied with 22 factors for predicting CVDs [19]. ML algorithms with different datasets, feature classifiers and accuracy rates were compared [20]. A method for automatic estimation of ML algorithms applied to CVDs was recommended in [21].

Fuzzy logic allows us to represent the common knowledge, mainly of the qualitative linguistic type, in a mathematical language through the theory of fuzzy sets and membership functions associated with them [22]. Fuzzy (or non-crisp) logic is a multivalued paraconsistent logic in which the true values of linguistic variables can be transformed into any real numbers between zero and one through probabilities. Therefore, it is employed to handle the concept of partial truth, where the truth value may range between entirely true and completely false. By contrast, in the Boolean (traditional or crisp) logic, the truth values of variables may only be the integer values zero or one. Fuzzy logic is based on the fact that people make decisions using imprecise and non-numerical information. This logic allows decisions based on intermediate degrees of compliance with a premise. Such logic is better suited to our real world, where our opinions are relative. Fuzzy logic is one of the best AI methods for coronary heart disease diagnosis. It is employed to predict disease occurrence with the help of linguistic variables and a membership function. Fuzzy logic uses linguistic variables, which usually enable us to measure by crisp numbers. Fuzzification is the next step for the identification of the variables. The rules are established with linguistic variables and their term sets which are the backbone of a fuzzy model called fuzzy inference systems.

A fuzzy model was proposed using membership functions (MFs) to find out the number of MFs affecting the outcome optimality and accuracy of a fuzzy model [23,24,25]. The performance of an adaptive neuro-fuzzy inference system (ANFIS) model is determined by how well the system parameters are chosen, the complexity they have and the type of training offered by ANNs [26]. An ANFIS method to classify the CVD degree was developed using seven factors along with the k-fold cross-validation method and the patient’s heart disease degree was successfully estimated with a 92.3% accuracy rate [27]. ANFIS controllers were utilized to compare the feedback from the output of electrocardiogram (ECG) signals, determining a control scheme for people who suffer from CVDs  [28]. A medical diagnostic system based on ANFIS and principal component analysis was investigated to forecast the CVD risk with a classification accuracy of 93.2% [29].

The gap in the investigation of CVDs is focused on enhancing the prediction accuracy using numerous factors with traditional classification methods [18,19,20]. Nevertheless, mainly the causes of CVDs are not known precisely. Age, BMI, cholesterol level, diabetes, eating habits, family history of heart problems, gender, high blood pressure, smoking, as well as an unhealthy and stressful lifestyle are the major factors affecting CVDs. In recent studies, the ANFIS and ML approaches were employed for predicting CVDs using factors such as age, BMI, cholesterol level (LDL/HDL), family history, F-glucose, gender, glucose level, high pressure, lifestyle, nationality, past medical history (PMH), red blood cell (RBC), smoking and stress level. As mentioned, some CVD factors are measurable, some are categorical and the response variable is also categorical. It is necessary to use the Gifi transformation method to balance the data and not oversimplify the complexity of the problem. This transformation includes categorical and measurable risk factors with non-linear interactions and converts the data into a measurable form. The prediction is based on previous learning and performs its duties best if the training data are not extrapolated [30]. It is possible to predict the patients who suffer the CVD. As a branch of AI, ML is increasingly utilized for predicting CVDs.

### 1.2. Contributions and Plan of the Article

Based on the complete bibliographical review presented above, we identified a gap that allows us to propose a methodology to improve predictive accuracy when detecting CVDs using numerous risk factors. We employ AI techniques based on ML, adaptive neuro-fuzzy and statistical approaches for the early prediction and classification of CVDs.

Consequently, the primary motivation for the present investigation is to provide timely medical treatment and diagnosis using an intelligent system based on current digital technologies. This system must besides effect efficient patient monitoring. Therefore, the main objective of the present investigation is to design and put into practice a system to improve predictive accuracy when detecting CVDs employing several risk factors.

Our contributions to the area can be summarized as follows:

- ML, ANFIS and statistical classification tools supported by the Gifi method are utilized to predict CVDs early in a more precise way.
- The effects of seventeen parameters on CVDs are investigated in depth using response surface methodology (RSM).
- The obtained findings are matched with the state-of-the-art studies comprehensively.
- Sensitivity analyses are carried out for ANFIS and SVR to determine the influence of significant factors such as age, BMI, glucose, cholesterol, RBC and HDL/LDL cholesterol levels on CVD.
- The results of statistical approaches with the Gifi method are given using statistical classification tools and linear discriminant analysis.
- The Nash–Sutcliffe model efficiency (NSE) coefficient is used to quantitatively describe and assess the model output’s predictive accuracy.
- We compare the capability of an adaptive elastic net logistic regression (AENLR) [31] and Gifi transformation with ML techniques (SVR, MARS, M5Tree and ANNs).

The plan of the present article is as follows. Section 2 points out the methodology used in the present investigation; Section 3 gives the results and findings of the present study. In Section 4, we discuss the performance of the applied approaches as well as some limitations of our study. In Section 5, conclusions related to the present investigation are provided.

## 2. Methodology

### 2.1. Dataset and Framework of the Study and Patients

The dataset was collected retrospectively from the medical record system of family medicine and cardiology clinics at a university hospital in Saudi Arabia, including 159 patients over the age of 16 who visited the cardiology clinic and complained of heart disease symptoms for over four months between 6 June 2020 and 10 October 2020. Each patient was tested for biometric measurements, ECG and bold lab works (such as F-glucose, HBA1c, cholesterol levels and RBC). In addition, every individual was asked about all other historical diseases that they had. Then, the diagnosis of the presence or absence of cardiovascular diseases for a patient was determined according to the expert opinion of the medical doctor based on the hospital records for each patient.

For this retrospective observational study, the data were collected with no names or identification (ID) numbers to preserve the confidential records after obtaining administrative permission from the university hospital. Thus, this research depends on clinical and laboratory data collection; no experimental interventions were needed or applied. Typically, most of the measurable data could be collected online. Nevertheless, we used retrospective data to avoid wasting time and costs. We decided on our full criteria set from the early beginning of our work, considering the demographic constraints in Saudi Arabia. We did not exclude or include any other criteria during the work. These criteria were determined by an expert medical consultant, one of the authors of the present article. As a statistical analysis, CVD prediction and classification were made with ML approaches and elastic net modeling. The MATLAB software was used for all computations.

The dataset is collected to (i) classify and determine the best predictors (covariates) during analysis; (ii) build different ML classifiers and employ them for achieving an adequate model; and (iii) provide an appropriate analysis regarding the transparency of classifiers and the reasoning process to improve medical physicians’ prediction accuracy. Table 1 describes the CVD dataset considering seventeen input variables corresponding to CVD risk factors, which play an essential role in CVDs and are specifically chosen by the experts in CVD and family medicine. Table 2 reports all the risk factors with their sources.

### 2.2. Gifi System for Data Transformation

The purpose of the Gifi method in this study is to convert categorical data into measurable data. The labels of the research’s ordinal or nominal factors contain some metric properties. To transform data with the Gifi method, scaling and linear combination methods are used together. We assign the ideal scale values to each factor class depending on the procedure optimizing criterion. Qualitative variables are converted to measured variables in the optimal scaling method. In contrast, the linear combination method converts multi-dimensional categorical data into one-dimensional continuous space by linearly combining their classes.

When the categorical variables have a high dimension, the linear combination method is more helpful [49]. Let $\left(s_{1},\ldots ,s_{m}\right)$ be an m×1 vector holding the number of classes for each factor and *p* signify the dimensionality of the analysis required. We code each variable $\delta_{l}$, for l∈{1,…m}, into the $n\times s_{l}$ matrix $H_{l}$.

Let *X* be the object score, represented by an n×p matrix (often p≤m). If $Y_{l}$ is the quantifying of the variable classes $\delta_{l}$, $H_{l}y_{l}$ indicates a modification or quantification for each of the *n* elements of the variable $\delta_{l}$. Objects in the same class acquire the same quantization without further requirements on $Y_{l}$.

In a homogeneity analysis, the quantization for each variable is gathered in the $s_{l}\times p$ matrices $Y_{l}$. As a result, for the variable $\delta_{l}$, $H_{l}y_{l}$ produces quantifications of the elements. For instance, we have that

$$\delta_{l}=\left[\begin{array}{c} a\\ b\\ a\\ c\\ c\\ a \end{array}\right]=\left[\begin{array}{c} Y_{1}\\ Y_{2}\\ Y_{1}\\ Y_{3}\\ Y_{3}\\ Y_{1} \end{array}\right]=\left[\begin{array}{c} 1\\ 0\\ 1\\ 0\\ 0\\ 1 \end{array}\;\;\begin{array}{c} 0\\ 1\\ 0\\ 0\\ 0\\ 0 \end{array}\;\;\;\begin{array}{c} 0\\ 0\\ 0\\ 1\\ 1\\ 0 \end{array}\right]X\left[\begin{array}{c} Y_{j1}\\ Y_{j2}\\ Y_{j3} \end{array}\right]=H_{l}y_{l},$$

where $H_{l}y_{l}$ denotes a single transformation generated by variable *j* on *n* objects. A homogeneity analysis minimizes a loss function given by $\sigma_{X;Y_{1},\ldots ,Y_{m}}=\left(1/m\right)\sum_{l=1}^{m}\mathrm{SS}\left(X-H_{l}y_{l}\right),$ where the sum of squares (SS) is the matrix elements. Under normalization, the loss function is reduced concurrently over object scores *X* and $Y_{l}$ using an iterative approach known as the alternating least squares algorithm; see [50,51,52,53,54] for more information on the Gifi transformation. Here, the categorical variables that need to be transferred to the Gifi systems are activity, gender, nationality, PMH, smoking and symptoms.

### 2.3. The Support Vector Machines Method

SVM is a powerful nonparametric ML approach that can predict and classify complex problems [55]. The method is effective for problems that have nonlinear relations between inputs and output variables. The input vector (*X* with observed values denoted by *x*) can be mapped into the output response using an *N* set of input ($X_{i}$) variables in SVR [56]. The nonlinear relation in SVR is defined using the expression $Y\left(x\right)=b+\sum_{j=1}^{N}\left(\omega_{1}-\omega_{N}\right)L\left(x,x_{j}\right)$, where $\omega_{1}-\omega_{N}$ are the weights used to link the input and output data and *b* is the bias. Here, $L(x,x_{j})$ denotes the kernel function that transfers the input data from real space into *N*-dimensional feature space. The kernel is determined commonly by utilizing the Gaussian radial basis function to define the nonlinear relations stated as $L\left(x,x_{j}\right)=\exp\left(-0.5{∥x-x_{j}∥}^{2}/\sigma^{2}\right)$, where σ is the kernel parameter. Here, γ is the value in the SVR procedure [57]. An optimization procedure determines the regression model to verify the unknown parameter weights using two slack variables, ζ and $\varpi^{*}$ namely, as

$$\begin{array}{cc} \mathrm{Min} & \frac{{∥\omega ∥}^{2}}{2}+D\sum_{j=1}^{N}\left(\varpi_{j}+\varpi_{j}^{*}\right),\\ \mathrm{ST}: & y_{j}-<\omega L\left(x,x_{j}\right)>-c,-c\leq \gamma +\varpi_{j},\;\omega L\left(x,x_{j}\right)>-y_{j}+c\leq \gamma +\varpi_{j}^{*},\;\varpi_{j}^{*},\varpi_{j}\geq 0; \end{array}$$

where γ is the residual used to control the predicted value Y(x) and the observed value denoted by *O*, when $\left|Y(x)-O\right|$ is less than γ and then the error is identified as zero. The Karush–Kuhn–Tucker conditions are applied and the optimum values of

$$\begin{array}{cc} \mathrm{Max} & -\frac{1}{2}\sum_{j,l=1}^{N}\left(\vartheta_{j}-\vartheta_{j}^{*}\right)\left(\vartheta_{l}-\vartheta_{l}^{*}\right)L\left(x_{j},x_{l}\right)+\sum_{j=1}^{N}\gamma (\vartheta_{j}^{*}-\vartheta_{j})-\sum_{j=1}^{N}y_{j}\left(\vartheta_{j}^{*}-\vartheta_{j}\right)\\ \mathrm{ST}: & \sum_{j=1}^{N}(\vartheta_{j}^{*}-\vartheta_{j})=0,\;0\leq \vartheta_{j}\leq D,\;0\leq \vartheta_{j}^{*}\leq D; \end{array}$$

are determined by employing the Lagrange relation to maximize the regression function, where $\vartheta_{j}^{*},\vartheta_{j}$ denote the corresponding multipliers. The SVR is approximated by

(1) $$\begin{array}{c} Y\left(x\right)=b+\sum_{j=1}^{N}\left(\vartheta_{j}-\vartheta_{j}^{*}\right)L\left(x,x_{i}\right). \end{array}$$

The three main parameters of the SVR approach (D,γ,σ) were presented in (1) and they must be defined in the modeling process.

### 2.4. Fuzzy Rules and Membership Functions

MFs can be conveniently defined and expressed by mathematical equations. Parameters (D,σ) are used to identify Gaussian MFs, where *D* and σ are the MF center and width. Additionally, there are operators called hedges, such as (very, quite, more or less) and connectives, to change their meaning in fuzzy terms. We consider that

(2) $${\mathrm{Gaussian}}_{x,c,\sigma }=\exp\left(-0.5{\left(x-c/\sigma \right)}^{2}\right),$$

(3) $$\mu \left(x_{5}\right)=\mu_{\mathrm{normal}}=\left\{\begin{array}{c} 0;\begin{array}{ccccc} \; & \; & & \mathrm{for} & x<1\;\begin{array}{cc} \mathrm{and} & \begin{array}{c} x>5.5; \end{array} \end{array} \end{array}\\ \exp\left(-\frac{1}{2}{\left(\frac{x-3.5}{1.9}\right)}^{2}\right),\begin{array}{cc} \; & \mathrm{for}\begin{array}{cc} \; & \end{array}\begin{array}{c} 1\leq x\leq 5.5; \end{array} \end{array} \end{array}\right.\;$$

where $x_{j}$ is the specific crisp input variable and $\mu (x_{j})$ is its membership degree. The membership degrees can be used to specify the fuzzy variables identified by the linguistic terms numerically. A Sugeno fuzzy rule-based ANFIS model assumes that IF $X_{1}$ is A, $X_{2}$ is B, $X_{3}$ is C and so on, THEN $Y_{j}=f_{j}\left(x_{1},\ldots ,x_{k}\right)=ax_{1}+bx_{2}+\ldots +kx_{k}+r_{j},$ where A, B and C are fuzzy terms in the premises part of the fuzzy rules, while $Y_{j}=f_{j}\left(x_{1},\ldots ,x_{n}\right)$ are crisp outcomes in the consequent part of the fuzzy rules illustrating the output of the fuzzy model in this work. Such rules are utilized in a loop (inner) of the model to establish the ANFIS and obtain crisp outcomes of CVD cases.

### 2.5. The ANFIS Approach

For the inference procedure, fuzzy reasoning is used to obtain crisp outcomes from the ‘IF–THEN’ rule. The fuzzy stage is the preliminary step of the inference system using fuzzified inputs in a specified universe. The firing strength of the rules is important and not all rules need to be triggered (fired) to achieve the requested outputs. In this study, the ANFIS models under consideration have only one output: the CVDs. The mean absolute error (MAE), mean bias error (MBE), the root of the mean square error (RMSE), desirability function (DF) and NSE are employed in the training/testing process to determine the error rate of the model. The MSE is calculated as

(4) $$\mathrm{MSE}=\frac{1}{n}\;\sum_{t=1}^{n}{\left(y_{t}-{\bar{y}}_{t}\right)}^{2},$$

where $y_{t}$ and ${\bar{y}}_{t}$ show the true output and forecasted value of the CVDs. The MSE shown in (4) produces a moderate error that may be preferable to one that usually has small errors and so the method can penalize large forecasting errors. The DF approach transforms the outcome values to a scale-free value, such as desirability, with values of 3, 4 and 5.

### 2.6. Response Surface Method for Factor Assessment and Sensitivity Analysis

The RSM is a mathematical and statistical optimization tool used to model and analyze problems in which several input factors influence the output response. The RSM solves the problems where the relation between input factors and the output response is unknown.

From the result analysis, the two modeling approaches (ANFIS and SVR) provide the robust capability for predicting the CVDs with the highest capability and lower error (high accuracy) among other modeling approaches. These two approaches are employed for the sensitivity analysis when effectively checking the primary influence of some input variables, such as age, BMI, glucose, cholesterol, RBC and LDL, on CVD prediction. The sensitivity analysis is computed using the differential predicted results as the marginal effect by increasing the input variables with Δx. The increasing input by Δx, as (x+Δx), is given in the models for data predictions. The mean of differences between the old prediction of input data with no increase and the new prediction obtained by input data being increased by (x+Δx) is compared for differential probability of CVDs stated as ${\mathrm{DF}}_{f}\left(\Delta x\right)=-\mathrm{mean}\left(Y\left(x+\Delta x\right)-Y\left(x\right)\right)$.

**Figure 1:**
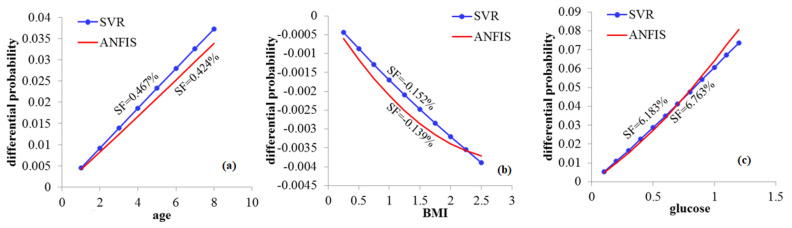
Plots of DFs for age (**a**), BMI (**b**) and glucose (**c**); see abbreviations in Nomenclature.

**Figure 2:**
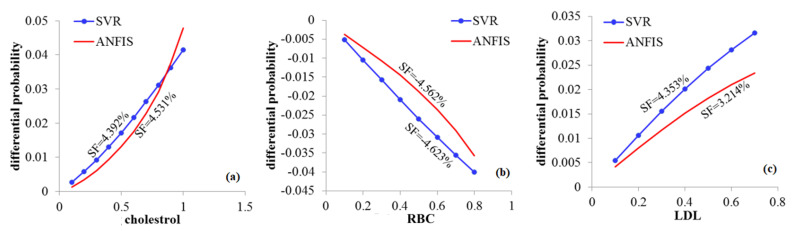
Plots of DFs for cholesterol (**a**), RBC (**b**) and LDL (**c**); see abbreviations in Nomenclature.

Considering the marginal effect of input variables using the differential method, the DP for several input variables, that is, age, BMI, glucose, cholesterol, RBC and LDL, are presented in Figure 1 and Figure 2 for the SVR and ANFIS methods. Based on the results presented, we define the sensitivity factor (SF) through the relation given by

$$\mathrm{SF}=\left(\frac{{\mathrm{DF}}_{f}\left(\Delta x_{j}\right)-{\mathrm{DF}}_{f}\left(\Delta x_{1}\right)}{\Delta x_{j}-\Delta x_{1}}\right)\times 100.$$

The SF represents the negative or positive effects of CVD inputs, showing the influence of inputs on the CVD. The highest SF indicates the influence of inputs that is highly sensitive.

### 2.7. Statistical Approaches for CVD Classification

Here, we not only present a wide range of soft computational approaches for CVD classification but also aim to compare them with statistical classification methods. For this purpose, some statistical classification tools such as linear discriminant analysis (LDA), quadratic discriminant analysis (QDA), k-nearest neighbor (kNN), naive Bayes (NB) and decision trees (DT) classifiers are used.

Different from classical classification algorithms, it has been proposed for the first time in the literature to determine the variables affecting the classification using AENLR analysis integrated with the the Gifi system data transformation method.

The AENLR model [31] is given by

(5) $${\hat{\beta }}_{\mathrm{AENLR}}={\mathrm{argmin}}_{\beta }\left\{-\sum_{i=1}^{n}\left\{y_{i}\log\left(\pi_{i}\right)+\left(1-y_{i}\right)\log\left(1-\pi_{i}\right)\right\}+\lambda_{1}\sum_{j=1}^{n}w_{j}\left|\beta_{j}\right|+\lambda_{2}\sum_{j=1}^{n}\beta_{j}^{2}\right\}.$$

By allocating small weights to large coefficients and big weights to small coefficients, adaptive weights are intended to assure regularization. The penalty term stated in (5) is formulated as

(6) $$P_{\mathrm{AENLR}}\left(\lambda_{1},\lambda_{2},\beta \right)=\lambda_{1}\sum_{j=1}^{n}w_{j}\left|\beta_{j}\right|+\lambda_{2}\sum_{j=1}^{n}\beta_{j}^{2}.$$

If we assume $\lambda_{2}>0$, then the expression established in (6) is strictly convex. Moreover, this penalty term can be written as

(7) $$P_{\mathrm{AENLR}}\left(\lambda_{1},\lambda_{2},\beta \right)=\lambda \left(\alpha \sum_{j=1}^{n}w_{j}\left|\beta_{j}\right|+\left(1-\alpha \right)\sum_{j=1}^{n}\beta_{j}^{2}\right).$$

where α∈(0,1) is an elastic net tuning parameter that controls the mixing between the $l_{1}$-norm and $l_{2}$-norm terms in the penalty. It is commonly recommended to use a relatively large value of α and then use it in 10-fold cross-validation to choose λ defined in (7).

After determining the variables contributing to the classification using AENLR, the results are compared with these variables using the five different classification procedures mentioned above.

### 2.8. Flowchart of the Methodology

**Figure 3:**
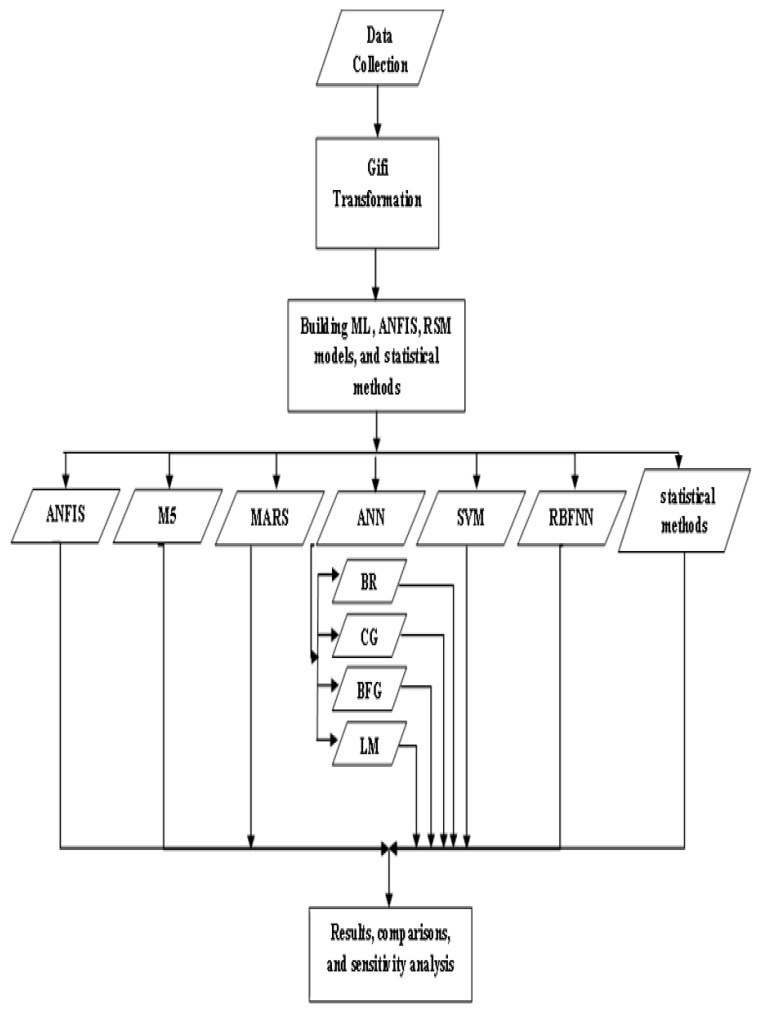
Flowchart of machine learning approaches for the prediction of cardiovascular diseases; see abbreviations in Nomenclature.

Figure 3 shows a scheme indicating that nine different AI approaches have been trained and tested for the dataset obtained from CVD patients. The performance of models is compared using the MAE, RMSE and MBE metrics.

## 3. Results and Findings

### 3.1. Exploratory Data Analysis

**Figure 4:**
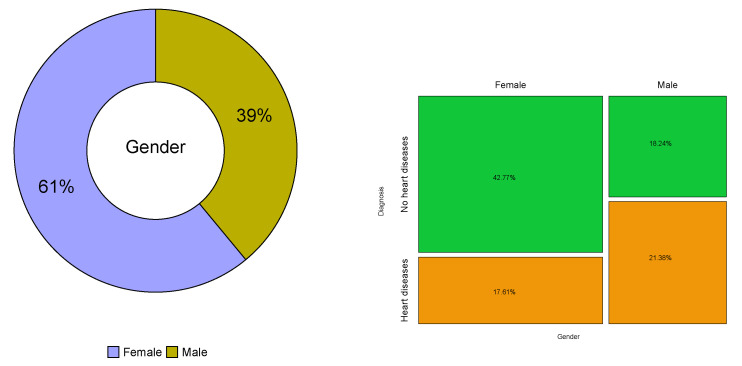
Distribution of the patients by gender (**left**) and gender versus diagnosis (**right**).

**Figure 5:**
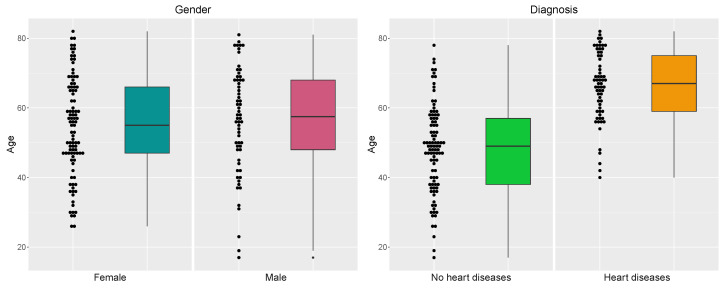
Distribution of the patients by age versus gender (**left**) and age versus diagnosis (**right**).

**Figure 6:**
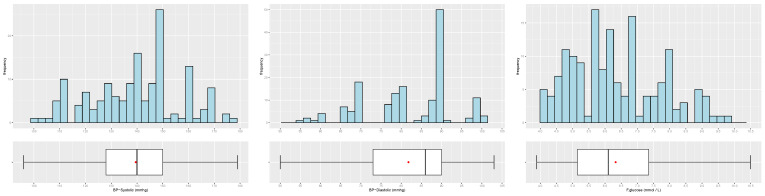
Distribution of the patients by systolic blood pressure (**left**), diastolic blood pressure (**center**) and F-glucose (**right**).

**Figure 7:**
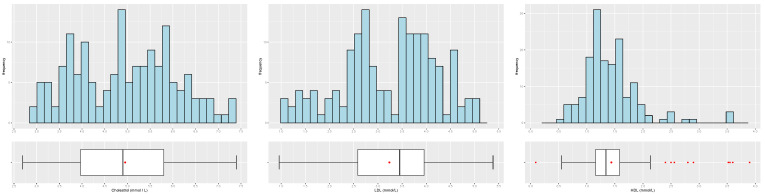
Distribution of the patients by cholesterol (**left**), LDL (**center**) and HLD (**right**).

**Figure 8:**
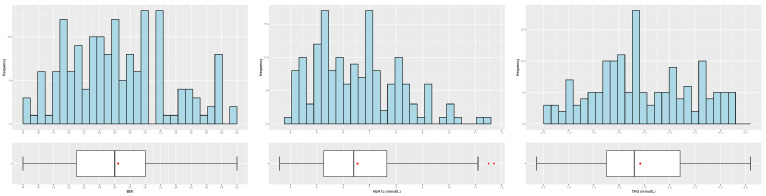
Distribution of the patients by BMI (**left**), HbA1c (**center**) and TAG (**right**); see abbreviations in Nomenclature.

Table 3 and Table 4 and Figure 4, Figure 5, Figure 6, Figure 7 and Figure 8 provide descriptive statistics of each variable under study.

Note that the gender ratio in the dataset is 61.01% (female) and 38.99% (male), with n=159 patients. The distributions of the continuous variables are mostly asymmetrical, with high variability, and present some outliers.

### 3.2. ANFIS for CVD Prediction

**Figure 9:**
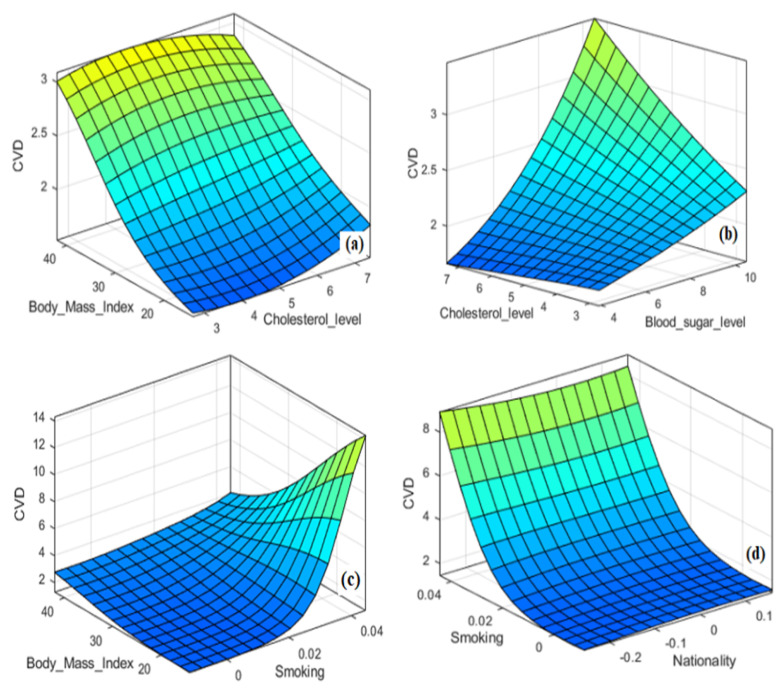
Three-dimensional relations of CVD with BMI, glucose level, smoking and nationality; see abbreviations in Nomenclature.

The ANFIS modeling approach is built based on the different learning capabilities of ANN algorithms. In this study, a hybrid learning algorithm is employed to derive the Sugeno ANFIS framework using the learning capability of the BPNN algorithm. As mentioned, the dataset was collected from a university hospital and the ANFIS approach predicts CVDs with crisp numerical outcomes. The ANFIS modeling approach includes the input variables, the fuzzy rules set, the MFs, the designed inference system and the defuzzification procedure for predicting CVDs. Different combinations of input and output relationships are trained/tested to reach the most suitable model for predicting the patient suffering the CVD. Figure 9a–d show the 3D relations of input parameters which are: (**a**) BMI-cholesterol level; (**b**) cholesterol level-glucose level; (**c**) BMI-smoking; and (**d**) smoking-nationality versus CVDs.

**Figure 10:**
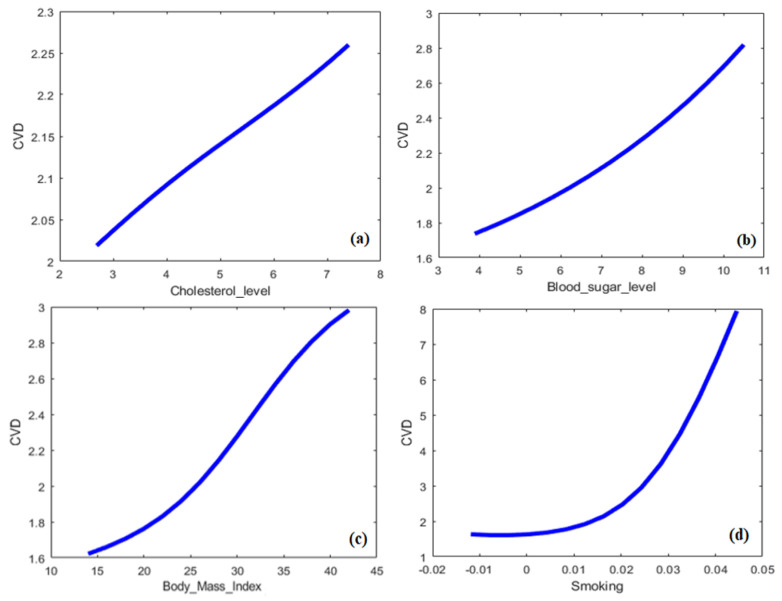
Two-dimensional relations of CVD with cholesterol level, glucose level, BMI and smoking; see abbreviations in Nomenclature.

Similarly, the 2D relations of some inputs, such as the cholesterol level, glucose level, BMI and smoking, versus the response (CVD), are presented in Figure 10a–d, respectively. As seen in Figure 9 and Figure 10, the relations of the input–output factors for CVDs are complex, ill-defined, unknown and remarkably nonlinear, which justifies the use of AI techniques. The 3D plots exhibit the full surface of the CVD output and the related input span. Hence, developing a mathematical model to solve this complex problem is difficult for decision-making. ML and ANFIS approaches can usually predict such complex problems. Hence, fuzzy methods and other intelligent modeling approaches, such as ANNs or hybrid intelligent systems, can be efficiently used with linguistic statements to solve imprecise and uncertain information [57] for predicting CVDs. Fuzzy and/or neuro-fuzzy modeling approaches can tell us more about the dynamics of CVDs by a set of linguistic associations with the help of input and output parameters. These associations use ‘IF–THEN’ rules to show the relationships of factors using variables related to linguistics and the corresponding terms. This ‘IF–THEN’ is the mapping of factors constituted from linguistic variables and terms, usually having two parts called antecedent and conclusion. The rule set is the backbone of an ANFIS. Gaussian memberships are utilized to detect the parameters and fuzzification process of the CVDs. The rule is utilized in a loop of the ANFIS model operating and obtaining crisp outputs for the classification of CVDs.

Our findings show that employing a small number of clusters (demonstrated by the rules) results in obtaining so many rules. In contrast, large cluster numbers generally produce fewer rules. Both are undesirable and must be avoided as they cause huge deviations in the prediction performance of the ANFIS model for CVD cases. Then, additional MFs do not increase the effectiveness of a fuzzy model [58].

### 3.3. Fuzzy Rules and Membership Functions

**Figure 11:**
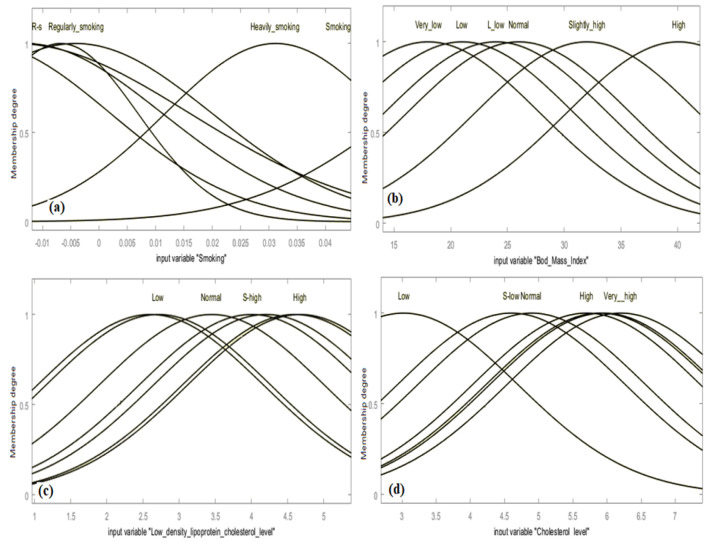
MFs for ANFIS model obtained for the estimation of CVDs; see abbreviations in Nomenclature.

Figure 11 shows the MFs which are fine-tuned for the input predictors: smoking ($X_{6}$), BMI ($X_{8}$), LDL cholesterol level ($X_{15}$) and cholesterol level ($X_{13}$). Different terms can be employed to identify fuzzy linguistic variables. For instance, the terms low, normal, high, very high; nonsmoker, average, highly smoking; low density, average and high HDL are the fuzzy linguistic terms used in this study.

A multi-input single-output ANFIS rule set for the prediction of CVDs can be exhibited as follows:

Rule 1:

IF ‘Gender is (woman)’ AND ‘Age is young (70.6)’ AND ‘Nationality is (Yemen)’…THEN ‘The CVD is 0.935’

⋮

Rule 7:

IF ‘Gender ($X_{1}$) is (man)’ AND ‘Age ($X_{2}$) is young (25.7)’ AND ‘Nationality (x_3_) is (Jordanian)’…THEN ‘The CVD is 0.0019’

### 3.4. The ANFIS Approach for CVD Prediction

As an inference procedure, fuzzy reasoning obtains crisp responses from the fuzzy ‘IF–THEN’ rules. In this study, the dataset is mixed, containing both data from categorical and continuous variables. To model the ANFIS approach and compare the results, the categorical data are transformed into continuous data using the Gifi system.

The input data was fuzzified to develop a fuzzy inference system in the specified universe. The second step was the MF formulation and the establishment of fuzzy rules. As seen in Figure 11a–d, in this work, we employ the Gaussian MFs, formulated based on the dataset obtained for the factors affecting the CVDs. Such rules are utilized in a loop (inner) of the model to establish the ANFIS and obtain crisp outcomes of CVD cases. Seven fuzzy rules were established based on the data available. Our model revealed that a small number of clusters (defined by rules) obtains too many rules. In contrast, many clusters generally caused a small number of rules. The RMSEs obtained from ANFIS models clearly show deviations in Table 5. Hence, the best ANFIS model producing the lowest RMSE is obtained when the number of MFs is seven. The fine-tuned MFs for the input variables: smoking ($X_{6}$), BMI ($X_{8}$), LDL cholesterol level ($X_{15}$) and cholesterol presence ($X_{13}$) are presented in Figure 11a–d. A fuzzy linguistic term set was established as ‘rarely smoking, regularly smoking, smoking, heavily smoking, not smoking at all’ and ‘very low, low, normal, slightly high, high’ for the fuzzy linguistic variables affecting the CVDs.

Gaussian MFs are utilized to identify the fuzzy linguistic variable ‘LDL cholesterol level ($X_{15}$)’. Its MFs are indicated by the fuzzy terms ’extremely low’; ’low’; ’normal’; ’high’; ’very high’; and enormously high’. The corresponding linguistic term ‘normal’ is mathematically stated in (2) and (3). The firing strength of each rule is necessary and it should be noted that not all the rules need to be fired to obtain the desired output. In this study, the ANFIS models under consideration have only one output: the CVDs. As seen in Table 6, eight ANFIS structures are developed and tested with several rules that were used to specify the CVD cases and minimize the prediction error. Initially, the error tolerance was set at 0.001 for the training process and 1000 iterations of the back-propagation multi-layer (BPML) algorithm were targeted. The MAE, RMSE and MBE approaches were used to assess ANFIS prediction performance. Additionally, the DF and NSE of the training and testing process were determined for an ANFIS structure; see Table 6.

The MAE, MBE, RMSE, DF and NSE are employed in training/testing processes to determine the error rate of the model and the results are given in Table 5. The ANFIS structure has average MSE, RMSE and MBE of 0.0165, 0.0679 and 0.0028, respectively, for the training process. As given in Table 5, the DF of the ANFIS model for the training process is the highest, with a value of 0.9829. The MAE, RMSE and MBE results show that the ANFIS approach reaches 0.0165, 0.0697 and 0.0028 error rates for the training process, respectively. For the testing process, the ANFIS approach obtains error rates of 0.2085, 0.3292 and 0.0062, respectively. The DF and NSE of the ANFIS approach are 0.9829 and 0.9656 for the training process, which are the highest rates among the other approaches.

The NSE coefficient is 0.9656, which quantitatively describes the predictive accuracy of the model output. The NSE for the training is high enough for the trained and tested ANFIS model. Similarly, the NSE coefficient is determined and used to describe and assess the predictive accuracy of model output quantitatively. The NSE is equivalent to the coefficient of determination (R${}^{2}$), so its range is between zero and one. An NSE coefficient close to one indicates a model with more predictive capability.

**Figure 12:**
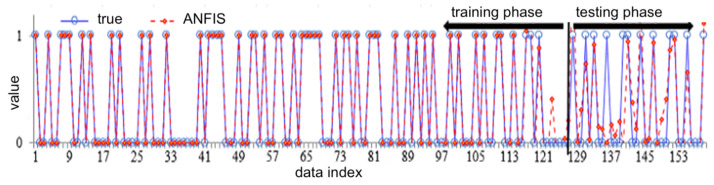
The true data points—blue—versus the training and testing outcomes—red color points and lines—of ANFIS approach; see abbreviations in Nomenclature.

Figure 12 shows the distribution of true data points versus the training/testing outcomes of an ANFIS structure for CVDs. This structure provides the lowest mean error (ME) and standard deviation (SD) among the other approaches, whose values are 0.0034 and 0.1603, respectively, showing its superior prediction capability with low uncertainty and robust approximation.

### 3.5. Elastic Net Modeling for CVD Prediction

We compare the capability of the AENLR and Gifi transformation with the ML techniques (SVR, MARS, M5Tree and ANNs such as ANN–BR, ANN–SCG, ANN–BFG, ANN–LM, RBFNN) employed for predicting the CVDs. The accuracy of these methods was investigated and is presented in Table 6. SVR, M5Tree and MARS are called probit models in statistics. They are the regression models where the dependent variable (*Y*) takes only two values: with ’1’ showing the cardiac disease and ’0’ indicating no cardio disease in our study. These modeling approaches predict the probability that, if a patient carries some specific characteristic, she/he may fall into one of these two specific classes.

We have a vector of covariates, including seventeen health-related variables influencing the CVD cases. In the present investigation, the covariates’ set includes gender ($X_{1}$), age ($X_{2}$), nationality ($X_{3}$), symptoms ($X_{4}$), PMH ($X_{5}$), smoking ($X_{6}$), activity ($X_{7}$), BMI ($X_{8}$), systolic blood pressure ($X_{6}$), diastolic blood pressure ($X_{10}$), F-glucose ($X_{11}$), HbA1c ($X_{12}$), presence of cholesterol ($X_{13}$), RBC ($X_{14}$), LDL ($X_{15}$), HDL ($X_{16}$) and ECG ($X_{10}$) and the response variable (Y) presence of CVD. Hence, the observed patients are categorized based on their predicted probabilities of being classified as a person who has a cardiac disease ‘1’ or does not have a cardiac disease ‘0’.

Eight different ML methods were trained and tested for the dataset of CVDs and the performances of the models were compared using MAE, RMSE and MBE. The results can be seen in Table 5. For checking the optimization of the responses, we employed DF. As seen in this table, the desirability of the SVR approach was found to be 0.9585 for the training. Similarly, the DFs of ANN–SCG and ANN–BFG are 0.9066 and 0.8987, respectively. In addition, the DF of the ANN–BR approach for the testing process is 0.7779, which is the highest among the approaches. Table 5 also shows that the other methods have slightly lower but closer DF values for the training and testing processes. For the ANN–SCG and ANN–BFG methods, the NSE coefficients are 0.8237 and 0.8079 for the training and testing processes, respectively. However, the other ML approaches have lower NSE coefficients for training and testing procedures. For instance, the ANN–BR has the highest testing coefficient of 0.6215, but the M5Tree and MARS have values of the NSE coefficient equal to 0.5730 and 0.5032, respectively.

As seen in Table 5, the SVR method gave 0.0387, 0.0389 and 0.0046 rates of error for the training process of CVD prediction using the MAE, RMSE and MBE methods, respectively. The desirability rate and NSE were found to be 0.9583 and 0.9195 for the training process. The testing process of the SVR method gave 0.2165, 0.2965 and −0.0041 error rates using MAE, RMSE and MBE, respectively. The DF and NSE are 0.7163 and 0.5382 for the testing process, respectively. In addition, ANN–SCG presents the best outcomes with 0.0847, 0.1232 and 0.0008 error rates of the training process using MAE, RMSE and MBE, respectively. The testing errors of the ANN–SCG approach are 0.2720, 0.3842 and −0.0344, considering MAE, RMSE and MBE, respectively.

As mentioned, the SVR is a powerful nonparametric approach of the ML method that is utilized for predicting the response of ill-defined problems with nonlinear relations between input and output factors. In the SVR approach, three main parameters (D,γ,σ) are identified in this work. Employing a method of trial-and-error, the three levels of parameters D∈{10,100,1000} and γ and σ are identified. Table 7 shows that the smallest RMSE values (a better response among other models) were obtained when D=10, γ=0.05 and σ=0.75. However, increasing *D* and σ, the RSME did not change significantly. Here, γ in the objective function of the SVR model is an effective parameter for predicting CVDs. The best outcome of the RMSE (0.0389) was obtained when γ was equal to 0.05.

**Figure 13:**
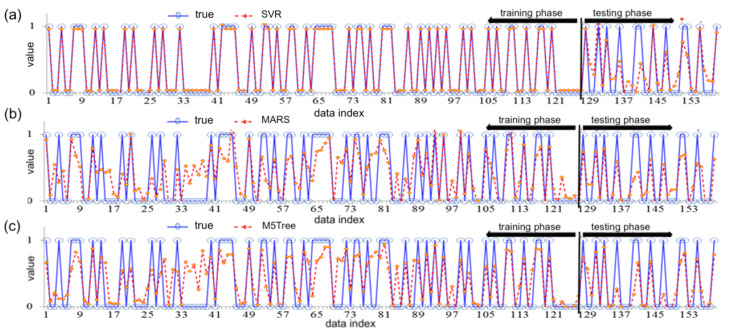
Plots of true data—blue—points used for the training and testing of the SVR (**a**), MARS (**b**) and M5Tree (**c**) methods and red points are the predicted outcomes; see abbreviations in Nomenclature.

Figure 13a shows the data distribution (blue color points and lines) of CVDs and the predicted data (red color points and lines) of CVDs obtained with training/testing processes of the SVR model. This model produces very close prediction outcomes of CVDs.

Figure 13b depicts the distribution of true data points (blue color points and lines) for training/testing processes of the MARS method versus the red color points and lines showing the distribution of the predicted CVD cases. In this figure, ‘1’ depicts the patients with cardiac problems and ‘0’ illustrates the patients who do not have cardiac problems.

The blue color points and lines in Figure 13c illustrate the distribution of true data points versus red color points and lines showing the distribution of the predicted CVD outcomes by the M5Tree method for the training and testing process.

**Figure 14:**
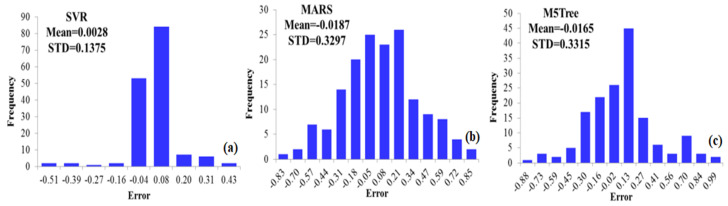
Distribution of the errors for the predicted and true data of CVDs in the training process using SVR (**a**), MARS (**b**) and M5Tree (**c**) of ML methods; see abbreviations in Nomenclature.

Figure 14a displays the frequency of errors for the predicted CVD cases, with mean=0.0028 and SD=0.1375 when the SVR approach is employed. The error ranges of the SVR approach are smaller, whereas the shape of its histogram is leptokurtic, showing that the findings are better and the approach is superior to the MARS (b) and M5Tree (c) models. For an SVR approach, Table 7 presents ME=0.0028 and SD=0.1375.

Figure 14b illustrates the distribution of the errors for the predicted CVD cases with mean=−0.0187 and SD=0.3297 (Table 8). Note that the M5Tree method is a piecewise regression model used for binary decisions. The linear regression functions are developed as the terminal nodes (leaves) to provide the relation between predictors for the causes of CVD risk in the M5Tree model.

Figure 14c shows the distribution of the errors for the predicted CVD cases with mean=0.0165 and SD=0.3315; see Table 8. Error ranges for prediction of the M5Tree method are high and the distribution is widespread.

As a statistical approach, the MARS method is used for predicting CVDs. In this method, the nonlinear regression is employed using the piecewise linear splines as a basic function, where a stepwise process is applied to explore the basic functions.

### 3.6. ANNs and Pattern Recognition

Multilayer ANNs are well known ML tools with the layers output, input and hidden. The ANNs provide a nonlinear mapping between the responses to the input parameters. We employ 1000 total iterations (epochs) for the training phase of the BPML algorithm with *M*-nodes (selected between 5 and 15) hidden to optimize the approach.

**Figure 15:**
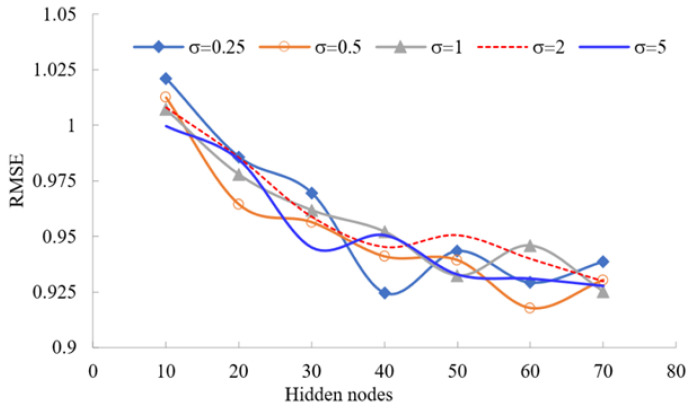
The RMSE for various hidden nodes of different RBFNN models; see abbreviations in Nomenclature.

We utilize a method of trial-and-error to give the best results when predicting message-passing neural network models. Moreover, we examine different optimization approaches for training the ANN models: Powell Beale conjugate gradient, BFG-BP and LM. The RMSEs for the training dataset using four BPML algorithms with various hidden nodes are presented in Figure 15.

The RBFNN is a fast-training algorithm that can be formulated efficiently to predict complex and ill-defined problems. An RBFNN model for predicting CVDs corresponding to various hidden nodes with different RBF parameters was compared in Figure 15 using RMSE. RBFNN is investigated for σ∈{0.25,0.5,1,2,5} and the number of hidden layers equal to 10, 20, 30, 40, 50, 60 and 70, with M+1 being unknown coefficients and *M* hidden nodes. Then, considering *M* as one of the main parameters in the RBFNN model, the *M*-centre of RBF is determined using the K-mean clustering approach. The best CVD prediction is calibrated with RBFNN when the hidden layers are 60 nodes and σ=0.5 by comparing the RMSE values. The lowest RMSE value is 0.9176, as depicted graphically in Figure 15.

The BR method provides stable results for different hidden nodes, but it is not a very accurate training approach compared to the others. The BFG is superior and provides accurate results for the training of ANNs compared to other optimization methods. The number of hidden nodes for the ANN model was selected as M>10. In the present study, it is set to M=11.

The ANN pattern recognition process is employed to find the regularities and similarities in a dataset using ML approaches. The similarities are investigated based on a statistical analysis of true historical data and the outcomes of algorithms. The best performance is selected from the iteration with a minimal validation error. After several training iterations, the error generally decreases. However, it may increase on the validation dataset as the network overfits the training data.

**Figure 16:**
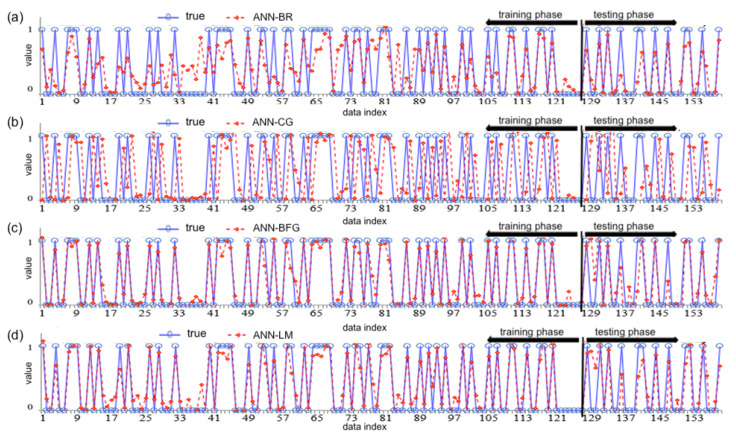
The true data—blue—points versus the predicted (red) data points using ANN–BR (**a**), ANN–CG (**b**), ANN–BFG (**c**) and ANN–LM (**d**) for the training and testing phases; see abbreviations in Nomenclature.

The outcomes of the ANN–BR algorithm are presented in Figure 16a. The figure shows the distribution of the true data points (blue points) versus red color points and lines showing the distribution of the predicted CVD outcomes with the ANN–BR approach for the training and testing phases. This algorithm employs the Jacobian matrix for minimizing the combination of weights and squared errors when determining the performance of responses, which produces a network that can oversimplify the optimization process. The ANN–BR network is trained for the inputs and outputs, whereas the best training performance is obtained at the 244 iterations.

From Figure 16b, note that the ANN–CG algorithm is one of the more successful methods for predicting CVDs.

The ANN–BFG is a deep learning algorithm. This is an alternative approach to the ANN–BFG methods for fast optimization. From Figure 16c, the algorithm produced successful outcomes with minimum error and SD for predicting CVDs in the training and testing phases. This figure illustrates the distribution of data points of the ANN–BFG approach. The blue color points and lines show the true data and the red color points and lines show the distribution of the CVDs predicted with this method.

The ANN–LM algorithm is also utilized for training/testing the CVD observation fitting problem and a two-layer feed-forward network is employed. The learning level of this algorithm is high and it is the fastest training algorithm. Moreover, the error rate is lower. Figure 16d shows the distribution of the true data points versus the training and testing outcomes of the ANN– LM algorithm depicted with red color points and lines of CVDs.

**Figure 17:**
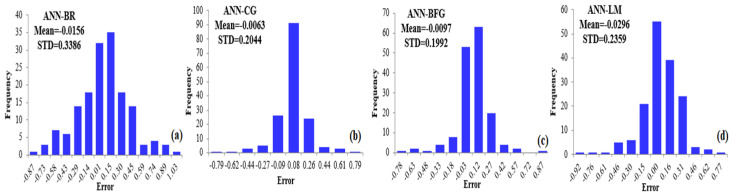
Distribution of error with CVD data using ANN–BR (**a**), ANN–CG (**b**), ANN–BFG (**c**) and ANN–LM (**d**) approaches; see abbreviations in Nomenclature.

Figure 17a shows the distributions of errors for the predicted CVDs with ME of −0.0156 and SD of 0.3386; see Table 9. The MSE and RMSE for the true and predicted CVD data are 0.1149 and 0.3389 for the ANN–BR algorithm, respectively. The ANN–CG algorithms search along the conjugate directions, which usually creates faster convergence than the steepest descent directions. The error level of this approach is found to be higher compared to the other algorithms.

Figure 17b shows the frequencies of errors for the predicted data of CVDs using the ANN–CG approach. Based on this algorithm, as reported in Table 10, the ME and the SD are −0.0063 and 0.2044, respectively. Therefore, the error level of this approach seems reasonable compared to the other soft comparing methods.

Figure 17c depicts the distribution of the predicted error. Hence, we obtain ME=−0.0097 and SD=0.2359; see Table 9. In addition, as presented in Table 11, the MSE and RMSE for the predicted CVDs are 0.0397 and 0.1994 for the ANN–BFG algorithm, respectively. This is one of the more successful algorithms for predicting CVDs.

Figure 17d shows the distribution of the predicted error of the ANN–LM approach, whereas the ME and SD are presented in Table 8. This optimization approach reveals ME=−0.0296 with SD=0.2359. The correlation and SD of ML models’ true and predicted data are presented in Table 9, which shows the difference between the targets and the output values of observation.

**Figure 18:**
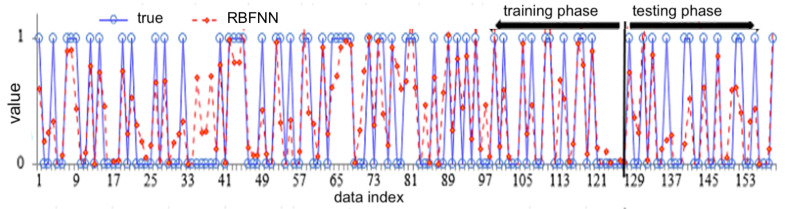
The true data points—blue—versus the training and testing outcomes—red points and lines—of RBFNN; see abbreviations in Nomenclature.

Figure 18 shows the true data points (blue) versus the training and testing outcomes (red color points and lines) of RBFNN approaches.

### 3.7. Response Surface Method for Factor Assessment

**Figure 19:**
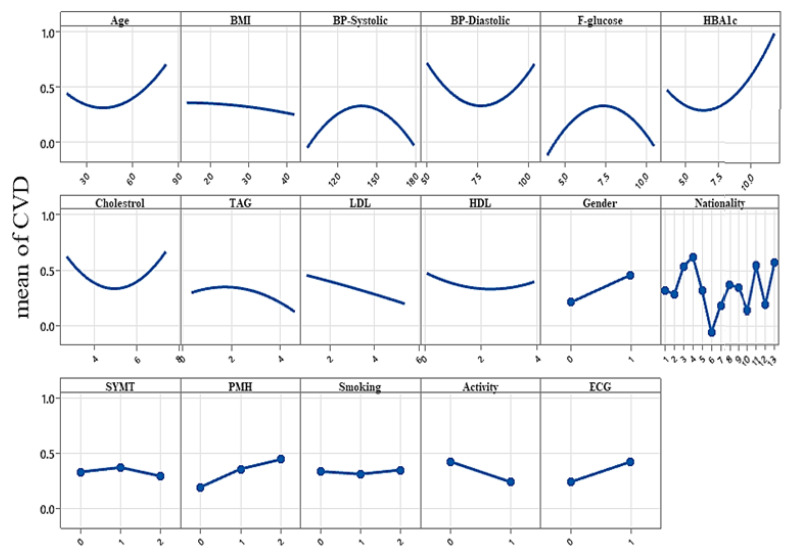
Plot of main effects for significant parameters on a CVD; see abbreviations in Nomenclature.

When a suitable approximation between the functional relationship of an output response (CVDs) and the nonlinear independent factors are determined, as we did for CVDs, an RSM-based polynomial approach might be a good approximation for a relatively small region problem. Figure 19 shows the plots of the main seventeen factors’ effect on the CVDs, constructed based on the RSM. The interactional relationship of measurable and categorical factors indicating the CVD risk is shown in this figure.

From Figure 19, note that the measurable factors related to pressure diastolic, age, F-glucose, HbA1c, HDL and the presence of cholesterol at a high level directly increase the risk of CVD. However, BMI, systolic blood pressure and RBC have a less significant effect on CVDs. In addition, an apparent CVD effect on gender is observed. It seems men suffer CVDs more than women. Shortness of breath slightly indicates a CVD problem. PMH is a strong indicator of CVD. Patients who have diabetes mellitus and hypertension can have a CVD. Even patients who have no abnormality detected may also have a CVD. Smoking also affects CVD negatively. Physical activity seems to have a direct positive effect on CVD, which reduces the risk drastically. ECG seems to be an essential categorical variable that identifies the patients who suffer from CVD. Our study covers patients from different nationalities, indicating that Jordanians suffer the least CVDs and Yemenis suffers the most. Consequently, gender, nationality, PMH, BMI, smoking, lifestyle, average glucose, LDL/HD, family history, high pressure and stress increase CVD risk. As a result, the predicted outcomes obtained with such formulations are distinct and can be matched using the dual and triple effect of parameters on CVD using the RSM.

### 3.8. Sensitivity Analysis

Our findings of SF show, as shown in Figure 1a, that age increase of one year increases the CVD probability by 0.467% and 0.424% according to SVR and ANFIS approaches, respectively. Decreasing the BMI by 0.5 units decreases the CVD risk by about 0.152% and 0.132%; see Figure 1b. Nonetheless, increasing the glucose also increases CVD probability by about 6.183% and 6.763 according to SVR and ANFIS approaches, respectively; see Figure 1c. Similarly, the increase in cholesterol increases the CVD probability by 4.392% and 4.531% according to SVR and ANFIS approaches, respectively; see Figure 2a. In contrast, every 0.1 unit decrease in RBC decreases the CVD probability by about 4.562% and 4.623%; see Figure 2b. However, every 0.1 unit increase in LDL increases the CVD probability by about 4.353% and 3.214 according to the SVR and ANFIS approaches, respectively; see Figure 2c.

### 3.9. CVD Prediction

**Figure 20:**
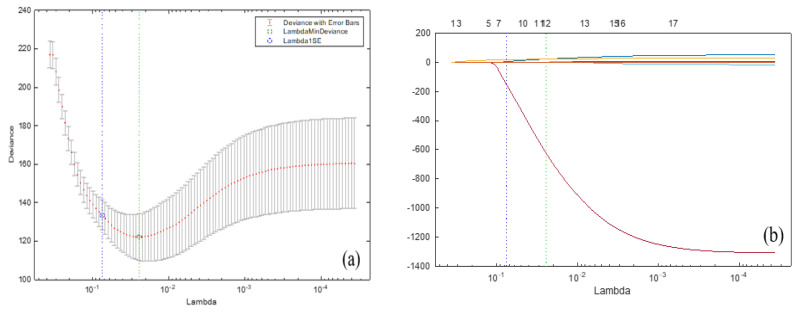
Plots of cross-validation deviance (**a**) and trace (**b**) of the elastic net fit, where λ with minimum error of cross-validation and α=0.9 is located at the green circle and dotted line; see abbreviations in Nomenclature.

As a function of the regularization parameter λ, with a green circle and a dashed line, Figure 20a emphasizes the minimum-deviance location. The blue-circled point has the smallest variance plus one SD. We use the parameter α=0.9 to encourage keeping groupings of strongly linked predictors rather than deleting all but one of them. The dotted line and green circle indicate the location of the least amount of the error employing a cross-validation method. Then, the location with the smallest error using a cross-validation method (plus one SD) is marked with a blue circle and a dotted line. The trace plot indicates non-zero model coefficients as a function of the regularization value. There are 17 curves in Figure 20b because there are 17 predictors in the linear model.

We indicate the point with a minimum error of a cross-validation method with the dotted line and blue circle (plus one SD) shown in Figure 20a. A trace plot with 17 curves is given in Figure 20b. As λ increases to the left, coefficients equal to zero are removed. We summarize the results in Table 11, which shows that the standard normal quartiles and the variables $X_{1}$ (gender), $X_{7}$ (activity) and $X_{17}$ (ECG), are the best choices with non-zero coefficients. According to the AELNR results, the factors affecting whether a person has cardiovascular disease or not were determined to be gender, activity and ECG. Therefore, using these coefficients for any statistical classification approach is recommended. The classification results obtained from the statistical methods are presented in Table 12.

## 4. Discussion

**Figure 21:**
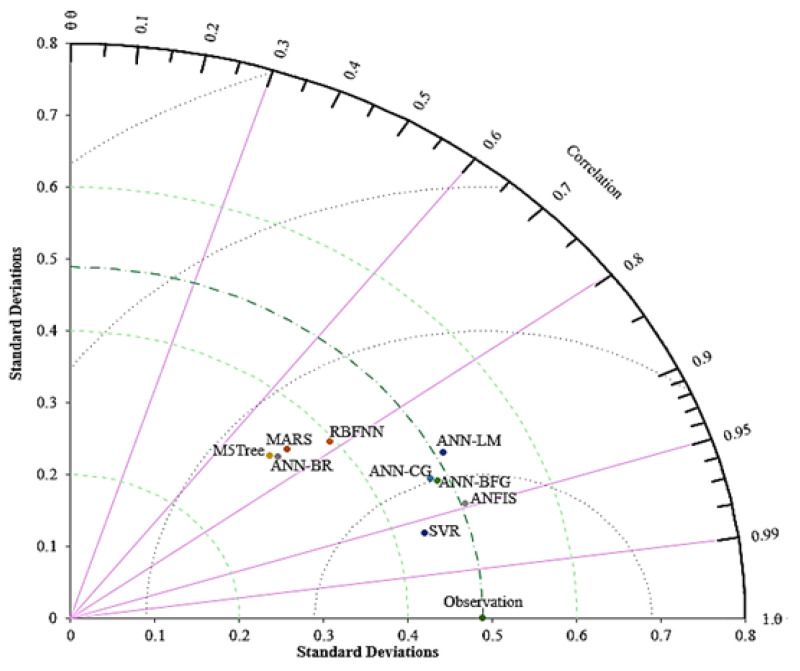
Taylor diagram for comparing the prediction capability of ML models; see abbreviations in Nomenclature.

The Taylor diagram is a mathematical scheme designed to graphically indicate the representations of patterns to match the models’ performance statistics simultaneously, that is, the correlation coefficient, SD and RMSE. These statistics can be plotted on a 2D graph to summarize the multiple aspects of models’ performance related to one another; see Figure 21. The findings clearly show that the SVR provides the highest correlation coefficient and SD of 0.4370 from the observed data compared to the other ML models. Similarly, the ANFIS approach depicted correlation coefficient and SD outcomes of 0.9471 and 0.4951, respectively, which is the closest value to the observed data. ANN–BFG and ANN–CG followed these two approaches. The Taylor diagram established for different ML models presented in Figure 21 provides better compression of ML methods when depicting the accuracy and SDs. Therefore, the model with the lightest observation point shows the highest trend. We use the SD of the observed and predicted values of the models as a measure of variability. We measure the variability as the radial distance utilizing the origin of the plot. The SD in Figure 21 represents the modeling uncertainty for the ML approaches. A model providing the smallest SD difference from the observed value among others exhibits a better prediction and low tension, providing a robust approximation.

The overall predictive ability and limitations of ML-based algorithms in CVDs were summarized in [11]. A comprehensive search study was designed for the prediction of these diseases [59]. Hence, the limitations can be organized as follows: firstly, the data are arbitrary because there are no standard guidelines for utilization. Hospitals have different data repository systems. In addition, clinical data are heterogeneous and usually imbalanced. Secondly, technical parameter-related data are usually not disclosed to the public, leading to high statistical heterogeneity. Some parameters are measurable and some are categorical. Third, criteria selection methods and procedures are arbitrary and heterogeneous. Fourth, we could easily classify the ML algorithms based on their performance. Fifth, several studies have reported different evaluation matrices.

Visits to the hospital during this investigation were restricted, especially since the time of compilation of the data coincided with the COVID-19 pandemic period. This is the reason for the relatively small number of samples used, which could be a limitation of this study.

We have held a sensitivity analysis for age, BMI, glucose, cholesterol, RBC and LDL. Though some ML algorithms and ANFIS approaches are robust, several studies have not reported a complete evaluation system of measurement. Then, some studies reported only the technical aspects without clinical aspects, likely due to a lack of clinician supervision. However, we determined our criteria and the data collection process under the supervision of an expert medical consultant. Table 13 presents the comparison of our findings with state-of-the-art methods. The ANN–LM algorithm showed the highest accuracy rate of 96.2%. The accuracy rate of the ANFIS approach is also high and is 94.7%.

## 5. Conclusions

CVDs correspond to the most common causes around the world related to mortality, affecting not only the heart and blood arteries but also heart failure, blood vessel diseases, stroke, arrhythmia, provoking a myocardial infarction. Determining the vital risk factors is crucial to intervening with the patient on time.

Relations between factors of CVDs are complex, ill-defined and nonlinear, justifying the use of artificial intelligence tools. These tools aid in predicting and classifying CVDs. In addition, mathematical/statistical models, such as those based on RSM, can solve complex problems in predicting CVDS when identifying 3D relations, which is not an easy task when making decisions.

ML can usually predict such complex problems and ANFIS approaches. Moreover, fuzzy logic and other intelligent models, such as ANNs or hybrid intelligent systems, can be used with linguistic statements to solve imprecise and uncertain information for predicting CVDs [57]. Moreover, fuzzy and/or neuro-fuzzy modeling approaches can tell us more about the dynamics of CVDs by a set of linguistic associations. In the present study, a comprehensive literature review was carried out using conventional ML and naive regression methods to detect well known risk factors of CVDs. To classify a patient as healthy or unhealthy, in our investigation, we used seventeen factors for predicting CVDs based on the M5Tree, SVR, MARS, feed-forward back-propagation, neural fitting, BR, SCG and ANFIS models.

We considered categorical and continuous variables, such as gender, age, nationality, PMH, BMI, smoking, lifestyle, F-glucose, cholesterol, average glucose, LDL/HDL, RBC, family history, blood pressure and stress levels. The Gifi system was used to convert the categorical data into continuous data. RSM was employed to determine the impacts of risk factors in 3D relations and very interesting conclusions were achieved.

MAE, RMSE and MBE were used to judge the performance of tools and approaches to simultaneously check the optimization of the outputs considering the desirability function. Moreover, the NSE coefficient was used to quantitatively describe and assess model prediction quality for the range between zero and one. In addition, a sensitivity analysis was performed to consider the marginal effects of factors such as age, BMI, glucose, cholesterol, RBC and LDL on CVDs, for two approaches. As detailed in the results and findings section, the age, BMI and glucose level were highly related to CVDs.

The ANFIS and SVR modeling approaches provided the highest prediction accuracy and tendency with the lowest error rates. The ANFIS prediction accuracy coefficient for the training process was 96.56%, followed by the SVR method with an NSE coefficient of 91.95%. The prediction accuracies of the other approaches were found as follows: ANN–CG 82.37% and ANN–BFG 80.79% for the training phase. The different ML approaches gave lower prediction accuracies for the NSE coefficient during the training. Furthermore, for the testing phase, the highest coefficient was found for the ANN–BR method at 62.15%, with the M5Tree, ANFIS and MARS models reaching values of 57.3%, 55.51% and 50.32%.

Based on linear discriminant analysis, the classification procedure obtained after the Gifi system application achieved a high classification performance, such as the successful ANN approaches. According to the results obtained from the prediction and classification using the linear discriminant analysis, gender, activity and ECG are the most significant variables that affect CVDs. As mentioned, our findings showed that employing a small number of clusters (demonstrated by the rules) resulted in obtaining so many rules. In contrast, large cluster numbers generally produce fewer rules. Both are undesirable and must be avoided as they cause huge deviations in the prediction performance of the ANFIS model for CVD cases. Then, additional membership functions do not increase the effectiveness of a fuzzy model [58].

This research covered a gap in CVDs as the researchers used numerous factors with traditional classification methods, which are imprecise indicators. For example, CVDs were classified [63] with traditional ML algorithms using 14 variables such as f-glucose, ECG and cholesterol from the healthcare dataset containing 76 attributes. The maximum accuracy rate of 91% was obtained by logistic regression. CVDs were estimated [64] with ML tools using 12 variables such as age, systolic and diastolic blood pressure. After making a variable selection using the correlation coefficient, the maximum accuracy rate they can reach with SVM was 78.84%. Using the UCI dataset containing 76 attributes, CVDs were classified [65] with many ML algorithms. After carrying out feature selection with the correlation map, the following accuracy rates were obtained for the traditional methods: 85% for LR, 81% for DT, 90% for SVM, 83% for RF, 90% for KNN and 84% for QDA. All these results showed that ANFIS combined with the Gifi data balance method has superior classification performance. Moreover, in terms of statistical methods, a high accuracy rate with only three variables after feature selection with AENLR is another outstanding result.

As the causes of CVDs are unknown, this uncertainty can be handled by employing fuzzy sets and systems to relate the factors and CVDs. The ML approaches help medical doctors enhance their diagnostic capability and accuracy, affecting patients’ prediction, quality of healthcare and efficacious medication prescriptions. Moreover, using such techniques has significance for healthcare centers, decreasing the time for medical exams, minimizing expenses in the clinical practice and enhancing practitioners’ efficiency [66].

Future research may improve statistical analysis, such as evaluating the computational complexity and ranking analysis of the models using statistical significance testing of post hoc methods. Moreover, applying other unsupervised ML methods, such as hierarchal clustering and anomaly detection with more data on nationalities, can significantly improve CVD prediction and classification. The methods derived in the present investigation are universal and based on artificial intelligence techniques. Therefore, they can be applied to practically all the areas where structured and unstructured data are available. Another future step might be developing an expert system to diagnose CVD patients.

## Figures and Tables

**Table 1:** The dataset characteristics and descriptions of variables; see abbreviations in Nomenclature.

Variable	Notation	Data Type	Coding and Description
Gender	$X_{1}$	Nominal	Female (1); Male (0)
Age	$X_{2}$	Continuous	Age of patents
Nationality	$X_{3}$	Nominal	SA = 1; EG = 2; SU = 3; YE = 4;
			IND = 5; JOR = 6; PAK = 7 PAL = 8; ETH = 9;
			CAN = 10; PHL = 11; TUN = 12; SY = 13
Symptoms	$X_{4}$	Nominal	SOB: shortness of breath; PMH: past medical history
PMH	$X_{5}$	Ordinal	PMH: Past medical history (DM: Diabetes mellitus = 1);
			HTN: Hypertension = 1;
			NAD: No abnormality detected = 0; DM and HTN = 3
Smoking	$X_{6}$	Ordinal	No = 0; Yes = 1
Activity	$X_{7}$	Ordinal	Low = 1; Normal = 0
BMI	$X_{8}$	Continuous	Body mass index
Systolic	$X_{9}$	Continuous	Systolic blood pressure
Diastolic	$X_{10}$	Continuous	Diastolic blood pressure
F-glucose	$X_{11}$	Continuous	Blood sugar (glucose) level
HbA1c	$X_{12}$	Continuous	Three-months average blood glucose (sugar) levels
Cholesterol	$X_{13}$	Continuous	Cholesterol test
RBC	$X_{14}$	Continuous	Red blood cell
LDL	$X_{15}$	Continuous	Low density lipoprotein
HDL	$X_{16}$	Continuous	High density lipoprotein
ECG	$X_{17}$	Ordinal	Electrocardiogram test; Normal = 1; Otherwise = 0
Diagnosis (CVD)		Nominal	Cardio disease = 1, No cardio diseases = 0

**Table 2:** Source of the CVD variables; see abbreviations in Nomenclature.

Variable	Source
Gender	Liao et al., 1997 [32]; Roeters van Lennep et al., 2002 [33]; Anderssen et al., 2007 [34]
Age	Anderssen et al., 2007 [34]; Dahlof, 2010 [35]
Nationality	Kurian and Cardarelli, 2007 [36]; Sibai et al., 2010 [37]
Symptoms	Hertz et al., 2020 [38]
PMH	Stampfer et al., 1988 [39]; Denes et al., 2007 [40]; Naghavi-Behzad et al., 2013 [41]
Smoking	Weycker et al., 2007 [42]; Dahlof, 2010 [35]
Activity	Twisk, 2000 [43]; Eisenmann, 2004 [44]
BMI	Weycker et al., 2007 [42]; Barroso et al., 2017 [45]
Systolic blood pressure	Weycker et al., 2007 [42];
Diastolic blood pressure	Denes et al., 2007 [40]; Weycker et al., 2007 [42]
F-glucose	Weycker et al., 2007 [42]
HbA1c	Weycker et al., 2007 [42]; Borg et al., 2011 [46]
Cholesterol	Dahlof, 2010 [35]
RBC	Kameneva et al., 1998 [47]; Dahlof, 2010 [35]
LDL	Weycker et al., 2007 [42]
HDL	Weycker et al., 2007 [42]
ECG	Dahlof, 2010 [35]; Rosiek and Leksowski, 2016 [48]

**Table 3:** Descriptive statistics of the continuous variable under study; see abbreviations in Nomenclature.

Variable	Notation	n	Mean	Standard Deviation	Median	Minimum	Maximum	Range	Skewness	Kurtosis
Age	$X_{2}$	159	55.21	14.7	56	17	82	65	−0.25	−0.61
BMI	$X_{8}$	159	26.45	6.82	26	14	42	28	0.37	−0.6
Systolic BP	$X_{9}$	159	139.5	18.74	140	96	179	83	−0.16	−0.52
Diastolic BP	$X_{10}$	159	81.8	11.56	86	50	103	53	−0.56	−0.37
F-glucose	$X_{11}$	159	6.33	1.45	6.1	3.89	10.5	6.61	0.48	−0.46
HbA1c	$X_{12}$	159	6.54	1.65	6.4	3.6	11.7	8.1	0.66	0.08
Cholesterol	$X_{13}$	159	4.95	1.16	4.9	2.69	7.4	4.71	0.07	−0.88
RBC	$X_{14}$	159	2.42	1.02	2.3	0.37	4.6	4.23	0.05	−0.83
LDL	$X_{15}$	159	3.24	1.02	3.45	0.96	5.38	4.42	−0.21	−0.58
HDL	$X_{16}$	159	1.44	0.55	1.34	0.09	3.9	3.81	1.83	5.44

**Table 4:** Descriptive statistics of categorical variables under study; see abbreviations in Nomenclature.

Variable	Notation	Values or Categories		
Gender	X1	Female	Male	
		61.01%	38.99%	
Symptoms	X4	SOB	PMH	NN
		61.01%	30.82%	8.18%
PMH	X5	DM	HTN	DM,HTN
		41.51%	33.33%	25.16%
Smoking	X6	NO	PAST	YES
		64.78%	10.06%	25.16%
Activity	X7	LOW	NORMAL	
		54.72%	45.28%	
ECG	X17	Normal	Change,STE,STD,SVT	
		49.06%	50.94%	
Diagnosis (CVD)	-	No heart diseases	Heart diseases	
		60.38 %	39.62%	

**Table 5:** The comparative results of the indicated model for training and testing phases; see abbreviations in Nomenclature.

Model	MAE	RMSE	MBE	DF	NSE
	Errors for Training Phase of Models				
SVR	0.0387	0.0389	0.0046	0.9583	0.9195
MARS	0.2700	0.3402	0.0021	0.6560	0.4383
M5Tree	0.2541	0.3382	0.0060	0.6782	0.4714
ANN–BR	0.2744	0.3557	0.0035	0.6399	0.4291
ANN–SCG	0.0847	0.1232	0.0008	0.9066	0.8237
ANN–BFG	0.0923	0.1253	0.0002	0.8987	0.8079
ANN–LM	0.1246	0.1564	0.0012	0.8620	0.7407
RBFNN	0.2185	0.2980	0.0021	0.7346	0.5455
ANFIS	0.0165	0.0697	0.0028	0.9829	0.9656
	Errors for Testing Phase of Models				
SVR	0.2165	0.2965	−0.0041	0.7163	0.5382
MARS	0.2329	0.2870	−0.1011	0.6928	0.5032
M5Tree	0.2002	0.3055	−0.1059	0.7493	0.5730
ANN–BR	0.1774	0.2622	−0.0914	0.7779	0.6215
ANN–SCG	0.2720	0.3842	−0.0344	0.7101	0.4198
ANN–BFG	0.2653	0.3677	−0.0489	0.7191	0.4339
ANN–LM	0.3500	0.4289	−0.1516	0.6629	0.2533
RBFNN	0.2553	0.3395	−0.0950	0.6857	0.4554
ANFIS	0.2085	0.3292	0.0062	0.7600	0.5551

**Table 6:** ANFIS models with MFs and training errors; see abbreviations in Nomenclature.

Modeling Approach	Number of Rules and MFs	Training RMSE	Number of Rules and MFs	Training RMSE
ANFIS	9	0.080	4	17.442
	11	11.119	6	6.444
	21	19.759	7	0.0697
	15	17.585	5	15.525

**Table 7:** The RMSE for the indicated value using the SVR method; see abbreviations in Nomenclature.

	D=500			D=1000			D=10		
	γ			γ			γ		
σ	**0.05**	**0.1**	**0.2**	**0.05**	**0.1**	**0.2**	**0.05**	**0.1**	**0.2**
0.50	0.0487	0.0924	0.1853	0.0487	0.0924	0.1853	0.0498	0.0996	0.1996
0.75	0.0486	0.0957	0.1917	0.0485	0.0958	0.1917	0.0389	0.0958	0.1907
1.00	0.049	0.0936	0.1753	0.0488	0.0935	0.1751	0.0725	0.1032	0.1838
1.50	0.0436	0.0934	0.1693	0.0484	0.0934	0.1691	0.1367	0.1512	0.1963

**Table 8:** The average prediction errors of ML approaches; see abbreviations in Nomenclature.

	SVR	MASR	M5Tree	ANN–BR	ANN–SCG	ANN–BFG	ANN–LM	RBFNN	ANFIS
ME	0.0028	−0.0187	−0.0165	−0.0156	−0.0063	−0.0097	−0.0296	−0.0174	0.0035
SD	0.1375	0.3297	0.3315	0.3386	0.2044	0.1992	0.2359	0.3063	0.1602

ME is mean error and SD is standard deviation.

**Table 9:** The correlation and SD of the true and predicted data of ML models; see abbreviations in Nomenclature.

Observation	SVR	MASR	M5Tree	ANN–BR	ANN–CG	ANN–BFG	ANN–LM	RBFNN	ANFIS	
Correlation	1	0.962	0.739	0.737	0.725	0.909	0.915	0.886	0.780	0.947
SD	0.489	0.437	0.348	0.335	0.327	0.469	0.476	0.499	0.394	0.495

SD: standard deviation.

**Table 10:** The true data versus the predicted data of CVDs using ML approaches; see abbreviations in Nomenclature.

True Outputs	SVR	MARS	M5Tree	ANN–BR	ANN–CG	ANN–BFG	ANN–LM	RBF–NN	ANFIS
1	0.96	0.923	0.662	0.704	0.999	1.052	1.083	0.603	0.999
0	0.04	0.550	0.208	0.380	0.015	0.014	−0.027	0.256	0.000
1	0.96	0.277	0.122	0.128	0.872	0.859	0.698	0.337	1.000
0	0.04	−0.048	0.179	0.060	0.044	0.088	−0.083	0.077	0.000
1	0.96	0.928	0.567	0.647	1.003	1.113	0.915	0.899	1.000
0	0.021	0.035	0.038	0.152	−0.082	−0.060	0.016	0.097	0.000
1	0.96	0.785	0.872	0.864	0.975	0.932	1.001	0.785	1.000
0	0.04	0.426	0.104	0.260	0.220	0.029	0.037	0.007	0.000
1	0.96	0.471	0.730	0.475	0.947	0.964	0.931	0.732	1.000
0	−0.03	0.059	0.047	0.032	−0.047	0.009	0.215	0.037	0.000
1	0.96	0.405	0.541	0.421	0.806	0.805	0.647	0.741	1.000
0	0.04	0.247	0.294	0.343	0.201	0.169	0.042	0.240	0.000

**Table 11:** Estimated coefficients for the variable indicated and their p-values; see abbreviations in Nomenclature.

Indicator		Estimated Value	Standard Error Value	*t* Statistic	*p* Value
*Y*-intercept		−4.828	3.862	−1.250	0.211
Gender	$X_{1}$	51.094	19.834	2.576	0.001
Age	$X_{2}$	0.041	0.031	1.301	0.193
Nationality	$X_{3}$	5.221	4.773	1.094	0.274
Symptoms	$X_{4}$	7.198	8.669	0.830	0.406
PMH	$X_{5}$	−2.703	10.288	−0.263	0.792
Smoking	$X_{6}$	−15.231	17.806	−0.855	0.392
Activity	$X_{7}$	−1311	492.99	−2.659	0.007
BMI	$X_{8}$	−0.022	0.050	−0.436	0.663
Systolic	$X_{9}$	0.021	0.028	0.736	0.462
Diastolic	$X_{10}$	−0.015	0.040	−0.383	0.702
F-glucose	$X_{11}$	0.201	0.414	0.485	0.627
HbA1c	$X_{12}$	0.113	0.413	0.274	0.784
Cholesterol	$X_{13}$	−0.163	0.361	−0.451	0.652
RBC	$X_{14}$	0.063	0.406	0.154	0.877
LDL	$X_{15}$	−0.023	0.479	−0.048	0.961
HDL	$X_{16}$	−0.640	0.544	−1.177	0.239
ECG	$X_{17}$	30.677	10.85	2.827	0.004

**Table 12:** The classification results obtained for statistical methods; see abbreviations in Nomenclature.

Method	TN	FN	TP	FP	CA	CER	Sensitivity	Specificity
LDA	80	6	57	16	86.16	0.1383	90.47	6.25
QDA	78	7	56	18	84.27	0.1572	88.88	7.29
kNN	79	7	56	17	84.91	0.1509	88.88	7.29
NB	77	6	57	19	84.27	0.1572	30.16	
DT	79	6	57	17	85.53	0.1446	82.29	26.98

LDA: Linear discriminant analysis; QDA: Quadratic discriminant analysis; kNN: k Nearest Neighbor classifier; NB: Naïve Bayes classifier; DT: Decision tree classifier; TP: True positive; FP: False Positive; TN: True negative; FN: False negative; CA: Classification accuracy; CER: Classification error rate.

**Table 13:** Comparison of our findings with state-of-the-art methods.

Methods	Accuracy (%)	Miss Rate (%)
Naive Bayes [60]	75.80	24.20
HRFLM [60]	88.40	11.60
Decision tree [60]	85.00	15.00
SVM [61]	88.00	12.00
Fuzzy-based ML	91.30	08.70
Framingham risk score [62]	687.04	12.96
Logistic regression [61]	89.00	11.00
Logistic regression [62]	86.11	13.89
ANFIS, our findings	94.70	5.30
ANN-LM, our findings	96.20	3.80
ANN-BFG, our findings	91.50	08.50

## Data Availability

Not applicable.

## Nomenclature

The following abbreviations are used in this manuscript:

AENLR	Adaptive elastic logistic net regression
ANFIS	Adaptive neuro-fuzzy inference system
ANN	Artificial neural network
ANOVA	Analysis of variance
AI	Artificial intelligence
BFG	Broyden–Fletcher–Goldfarb–Shanno quasi-Newton back propagation
BMI	Body mass index
BPML	Backpropagation multiple layer
BPNN	Back propagation in neural network
BR	Bayesian regularization
CVD	Cardiovascular disease
DF	Desirability function
DP	Differential probability
DT	Decision trees
ECG	Electrocardiogram
EEG	Electroencephalogram
FFB	Feed forward back propagation
FIS	Fuzzy inference system
HbA1c	Glycated hemoglobin
HDL	High density lipoprotein
kNN	k-nearest neighbor
LDA	Linear discriminant analysis
LDL	Low density lipoprotein
LM	Levenberg–Marquardt
MAE	Mean absolute error
MARS	Multivariate adaptive regression splines
MBE	Mean bias error
ME	Mean error
MESA	Multiple ethnic studies of atherosclerosis
MF	Membership functions
ML	Machine learning
NB	Naive Bayes
NSE	Nash–Sutcliffe efficiency
PMH	Past medical history
QDA	Quadratic discriminant analysis
RBC	Red blood cell
RBFNN	Radial basis functions neural networks
RSM	Response surface methodology
RMSE	Root of the mean square error
SCG	Scaled conjugate gradient
SD	Standard deviation
SF	Sensitivity factor
SS	Sum of squares
ST	Subject to
SVM	Support vector machines
SVR	Support vector regression
WHO	World Health Organization
